# Gene Regulatory Network Analysis of Post-Mortem Lungs Unveils Novel Insights into COVID-19 Pathogenesis

**DOI:** 10.3390/v16060853

**Published:** 2024-05-27

**Authors:** Ryan Bloomquist, Ashis K. Mondal, Ashutosh Vashisht, Nikhil Sahajpal, Kimya Jones, Vishakha Vashisht, Harmanpreet Singh, Jaspreet Farmaha, Ravindra Kolhe

**Affiliations:** 1 Department of Pathology, Medical College of Georgia, Augusta University, Augusta, GA 30909, USA; rbloomquist@augusta.edu (R.B.); amondal@augusta.edu (A.K.M.); avashisht@augusta.edu (A.V.); kjones1@augusta.edu (K.J.); vvashisht@augusta.edu (V.V.); hsingh1@augusta.edu (H.S.); jfarmaha@augusta.edu (J.F.); 2 School of Medicine, University of South Carolina, Columbia, SC 29209, USA; 3 Greenwood Genetic Centre, Greenwood, SC 29646, USA; nsahajpal@ggc.org

**Keywords:** COVID-19, host cellular responses, interleukin-17 signaling, inflammatory response, innate immunity, miRNA microarray

## Abstract

The novel coronavirus disease 2019 (COVID-19), caused by the severe acute respiratory syndrome coronavirus 2 (SARS-CoV-2), has emerged as one of the most significant global health crises in recent history. The clinical characteristics of COVID-19 patients have revealed the possibility of immune activity changes contributing to disease severity. Nevertheless, limited information is available regarding the immune response in human lung tissue, which is the primary site of infection. In this study, we conducted an extensive analysis of lung tissue to screen for differentially expressed miRNAs and mRNAs in five individuals who died due to COVID-19 and underwent a rapid autopsy, as well as seven control individuals who died of other causes unrelated to COVID-19. To analyze the host response gene expression, miRNA microarray and Nanostring’s nCounter XT gene expression assay were performed. Our study identified 37 downregulated and 77 upregulated miRNAs in COVID-19 lung biopsy samples compared to the controls. A total of 653 mRNA transcripts were differentially expressed between the two sample types, with most transcripts (472) being downregulated in COVID-19-positive specimens. Hierarchical and PCA K-means clustering analysis showed distinct clustering between COVID-19 and control samples. Enrichment and network analyses revealed differentially expressed genes important for innate immunity and inflammatory response in COVID-19 lung biopsies. The interferon-signaling pathway was highly upregulated in COVID-19 specimens while genes involved in interleukin-17 signaling were downregulated. These findings shed light on the mechanisms of host cellular responses to COVID-19 infection in lung tissues and could help identify new targets for the prevention and treatment of COVID-19 infection.

## 1. Introduction

The lung is one of the most extensively studied organs in the context of the COVID-19 pandemic, as it serves the primary sites of infection and a frequent site of organ failure [1]. The virus spreads through respiratory droplets, and due to the high density of angiotensin-converting enzyme 2 (ACE2) receptors (binding partner of SARS-Cov2’s spike protein) in nasal epithelium, it can be easily transmitted to and colonize the lower respiratory tract [2]. After infection is established, the host response progresses from viral entry and early infection to an early immune response marked by macrophages and dendritic cells, and a late immune response with cytotoxic T cells. If the host is unable to clear the infection, they may progress to a hyperinflammatory stage characterized by a cytokine storm, followed by multiorgan dysfunction and ultimately organ failure before death [3]. Current evidence suggests that these immunopathological responses are not solely determined by host characteristics and disease severity, but also exhibit distinct characteristics based on the type of organ or tissue affected. Specifically, variations in immunopathology are observed in different organs such as the lungs, heart, and stomach, as determined by relevant biological parameters [4].

Considering the central role played by lungs in establishment and pathology of COVID-19 infection, understanding the molecular processes taking place in lungs during severe and critical COVID-19 infections could be crucial in developing life-saving therapies. However, despite this rationale, only a limited number of studies have investigated the molecular mechanisms underlying COVID-19 in clinical lung tissues. Some studies have shown that the COVID-19 virus can repress the type IFN I response, which is an important part of the body’s antiviral defense mechanism [5]. However, cultured alveolar epithelium exposed to the virus has demonstrated that SARS-CoV-2 infection is unable to suppress downstream type I IFN signaling, and certain proteins, such as IRF7, IFIT1, and STAT 1/2, can help induce the first line of antiviral type I IFN response [6,7]. Other studies have used targeted immunohistochemical analysis and multi-omics approaches to investigate the molecular changes in COVID-19-infected lung tissues. In targeted immunohistochemical analysis, COVID-19 biopsy tissue was found to display significantly higher IL-4 tissue expression and Sphingosine-1 than in H1N1- and non-infected lung, but these data were limited to M2 macrophages [7]. Additionally, some studies have identified cytokine signaling and upregulation of certain genes, such as NFKB1, and STAT1, in COVID-19-infected tissue [8,9]. Another valuable dataset has been created by performing single-cell and single-nucleus RNA sequencing on various organs of 32 postmortem COVID-19-infected patients [10]. Here, COVID-19 alveolar tissue was found to be enriched with IFNα and IFNγ response genes and oxidative phosphorylation pathways. Here, highly infected tissue displayed upregulated IFIT1, IFIT3, IDO1, GZMB, LAG3, NKG7, and PRF1. Furthermore, SARS-CoV-2+ myeloid cells expressed TNFAIP6, CXCL11, CCL8, ISG1, and GBP5. Conversely, TNF, IL2, STAT5, and TGFβ signaling as well as apical junction/hypoxia pathways were downregulated. 

A more integrated approach might be to undertake a combined evaluation of mRNA and miRNA expression profiles of COVID-19 patients to gain insights into the intricate regulatory networks underlying the host response to the infection. MicroRNAs (miRNAs) are a class of small endogenous RNA molecules that play a crucial role in regulating various physiological processes within cells, including the maintenance of endothelial homeostasis, which is critical in the context of vascular diseases [11]. Aberrant expression of miRNAs has been linked to respiratory syndromes and pulmonary diseases caused by viral infections [12,13]. With regards to the current COVID-19 pandemic caused by SARS-CoV-2, most of the studies investigating miRNAs in association with COVID-19 have relied on computational miRNA prediction analyses [14,15]. However, to date, there have been no reports on the expression analysis of miRNAs and mRNA in lung biopsies of COVID-19 patients. 

In this study, we aimed to explore the transcriptome profiles of patients who succumbed to severe COVID-19 infection compared to those who died from unrelated causes. To achieve this, we collected biopsy specimens and performed comprehensive analyses of both regulatory miRNA and mRNA expression. Notably, this investigation represents one of the earliest attempts to analyze miRNA and mRNA simultaneously in COVID-19 patients at an organ level, enabling us to identify potential regulatory interactions. Our comparative analysis revealed distinct differences in miRNA and mRNA expression patterns between postmortem lung biopsy specimens of COVID-19-positive and COVID-19-negative lungs. Moreover, our computational analysis provided valuable insight into the cellular activities, signaling pathways, and regulatory mechanisms that are involved in the viral host response during acute COVID-19 deaths. Our findings shed light on new avenues for the development of therapeutic interventions in the fight against COVID-19.

## 2. Materials and Methods

### 2.1. Sample Collection

The present study describes the acquisition of five postmortem lung biopsy samples from patients who were treated for COVID-19 at Augusta University Medical Center (AUMC) in Augusta, GA, USA. In order to assess the specificity of miRNA deregulation as a result of SARS-CoV-2 infection, a control group consisting of seven postmortem lung samples was also collected from patients who had died due to other causes that did not involve lung injuries. The use of this control group enabled us to determine the specificity of miRNA deregulation due to SARS-CoV-2 infection and to eliminate the potential influence of tissue type on miRNA expression variation. The specimens were procured via postmortem dissection and subjected to fixation with formalin to prevent degradation of nucleic acids. Following fixation, the specimens were sectioned into slices 8 μm in thickness and subsequently affixed to a glass slide for further analysis.

### 2.2. Total RNA Isolation

The isolation of total RNA from formalin-fixed paraffin embedded (FFPE) lung tissue was performed utilizing the Qiagen (QIAGEN Inc, Valencia, CA, USA) miRNAeasy kit (217504), following the manufacturer’s protocol. A DNase treatment step was implemented during the isolation of total RNA to remove any residual DNA carryover. The quantity of the total RNA samples was determined utilizing an ultraviolet spectrophotometer (Nanodrop, Thermo Scientific, Pittsburgh, PA, USA). Total RNA samples isolated from FFPE samples were utilized for microRNA analysis and NanoString’s nCounter XT Gene Expression Assay.

### 2.3. miRNA Microarray Analysis

The quality of the RNA sample was assessed using the RNA integrity number, which was determined through analysis with an Agilent BioAnalyzer equipped with an RNA6000 Pico Chip. The miRNA microarray analysis was performed using an Affymetrix GeneChip^®^ miRNA 4.0 array at the Integrated Genomics Core of Augusta University, GA, USA. The labeling of 250 ng of total RNA with biotin was achieved using the FlashTag Biotin HSR RNA Labeling Kit from Thermofisher, following the manufacturer’s protocol. The labeled samples were then hybridized to the GeneChip miRNA 4.0 array, which included 1908 mature and 1255 premature miRNA. Array hybridization, washing, and scanning were carried out following the manufacturer’s instructions, and the resulting data were obtained in the CEL file format. The CEL files were then imported into Partek Genomic Suites version 7.0 using the standard import tool and subjected to RMA normalization for further analysis.

### 2.4. NanoString’s nCounter^®^ Host Reponse Panel

Nanostring’s proprietary nCounter^®^ Host Response Panel was employed to precisely measure mRNA expression levels in this study. This comprehensive panel consisted of 773 carefully selected genes, encompassing various aspects of the host immune response to infectious diseases. To ensure accurate data normalization, the panel also featured 12 internal reference genes (Table S1).

The nCounter^®^ Host Response Panel spanned five pivotal immune functional themes, each containing a substantial number of genes. The adaptive immune response pathway, for instance, comprised 483 genes, while the homeostasis pathway included 282 genes (Table S2). Additionally, the panel addressed host susceptibility with 26 genes, innate immune cell activation with 567 genes, and the interferon response with 288 genes.

### 2.5. NanoString’s nCounter XT Gene Expression Assay

NanoString’s nCounter (NanoString Technologies, Inc. 530 Fairview Ave N, Seattle, WA, USA) technology was used to analyze the comparison of host response gene expression between COVID-19 (+) and COVID-19 (−) groups. This technology is based on digital detection and direct molecular barcoding of individual target molecules, using a unique probe pair for each target of interest. The probe pair consists of a color-coded reporter probe, which carries the visible signal on its 5′ end, and a capture probe, which carries a biotin moiety on the 3′ end. A total of 300 nanograms of RNA with an OD260/280 ratio between 1.7–2.2 were subjected to hybridization with a reporter and capture code set (HS host response) at 65 °C for over 12 h. Excess probes were eliminated using a two-step magnetic bead-based purification method on an nCounter instrument. The resulting purified target–probe complexes were eluted off the beads, immobilized on the cartridge, and aligned for data collection. Data collection was performed using epifluorescence microscopy and CCD capture technology on an nCounter instrument, generating hundreds of thousands of target molecule counts. Digital images were processed within the nCounter instrument, and the reporter probe counts were tabulated in a comma-separated value (CSV) format. The CSV file was analyzed with NanoString’s free nSolver™ Analysis Software V.4 (NanoString Technologies, Inc. 530 Fairview Ave N, Seattle, WA, USA).

### 2.6. Functional Enrichment Analysis 

To understand the regulatory interactions between miRNA and mRNA, we used miRDIP (Version 5.3.0.1), a web-based platform that allows for the identification of bidirectional overlapping interactions between miRNAs and mRNAs in Homo sapiens. A total of 40 mRNAs showing the highest negative and positive fold differential expression in COVID-19 (+) versus COVID-19 (−) lung biopsy specimens were queried against a set of 113 significantly differentially expressed miRNAs. To enhance the reliability of our results, we used miR target predictions derived from two different databases, namely MBStar and miRbase, with a medium confidence threshold level; i.e., interactions were ranked in the top one-third (33.33%) of the predicted interactions reported [16]. 

Further, the Search Tool for Retrieval of Interacting Genes (STRING) database, which integrates both known and predicted protein–protein interactions (PPIs), was used for exploring the functional implications of our findings (https://string-db.org/ accessed on 1 March 2024). The active interaction sources including text mining, experiments, databases, and other co-expression analysis were used by STRING to construct the PPI networks, and the outcomes are represented as nodes and edges. The nodes correspond to the proteins, and the edges represent the interactions. Specifically, differentially expressed mRNAs were queried for proteins with values/ranks against Homo sapiens with log2-fold change input. The resulting network outcomes were visualized as nodes and edges, with nodes corresponding to the proteins and the edges representing the interactions.

Finally, we manually analyzed the Kyoto Encyclopedia of Genes and Genomes (KEGG) pathways for the highest associations with differentially expressed gene sets.

### 2.7. Statistical Analysis

To visualize the partition among the groups and identify the major sources of variation within the experiment, we conducted principal component analysis (PCA) Partek (Partek^®^ Flow^®^ software, version 9.0.20.0622, Copyright © 2020, Partek Inc., St. Louis, MO, USA). Additionally, the differential expressions were calculated using ANOVA of Partek Package and heat maps were generated by hierarchical clustering of miRNA. The results were normalized using robust multichip averages. T-tests were used to determine whether there was a significant difference (2.5 fold) for miRNA expression between the COVID-19 (−) and the COVID-19 (+) groups. The *p*-value (0.05) was taken as significant.

## 3. Results

### 3.1. COVID-19 Patients and Control Demographics

In this study, a cohort of 12 samples was subjected to analysis, comprising 7 males and 5 females, with an age distribution ranging from 33 to 100 years (Table 1). Among these specimens, 5 were diagnosed with COVID-19, encompassing 4 males and 1 female, aged between 40 and 80 years. All COVID-19 cases resulted in mortality attributed to COVID-19-induced pneumonia. The control group samples predominantly exhibited pathology consistent with atherosclerotic cardiovascular disease and complications arising from diabetes, contributing to their fatal outcomes. Also, one control was included as duplicate sample in Nanostring analysis but not in the miRNA analysis.

### 3.2. miRNA Expression Alterations in Lung Biopsy Specimens of COVID-19 Patients

**Figure 1:**
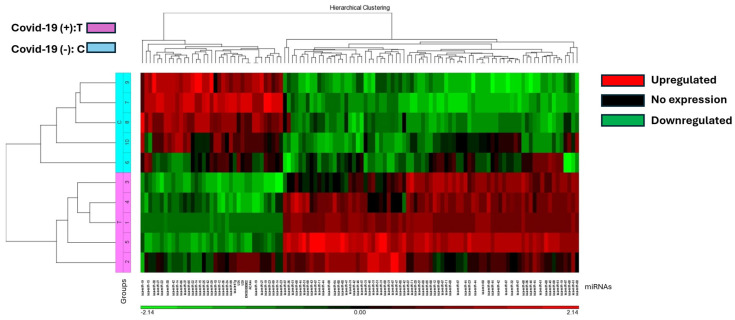
The heat map illustrates miRNA expression patterns in COVID-19 positive (T) and negative (C) lung biopsies. Five samples from each group were analyzed using the Affymetrix array system. miRNAs are represented as columns, while samples are depicted as rows. Expression levels are color-coded: red indicates higher expression, black indicates no expression, and green indicates lower expression. COVID-19 positive samples are denoted in pink, while negative samples are highlighted in blue.

The study identified 37 miRNAs that were downregulated and 77 miRNAs that were upregulated in COVID-19 (+) lung biopsy specimens compared to COVID-19 (−) lung biopsy specimens, with a significant difference of 2.5-fold miRNA expression and a *p*-value of 0.05 (Table S3). Hierarchical cluster analysis was used to generate a heatmap of the differentially expressed miRNAs using the Partek Package (Figure 1). The analysis revealed a distinct split between those who died from COVID-19 and those who died from other causes. 

**Figure 2:**
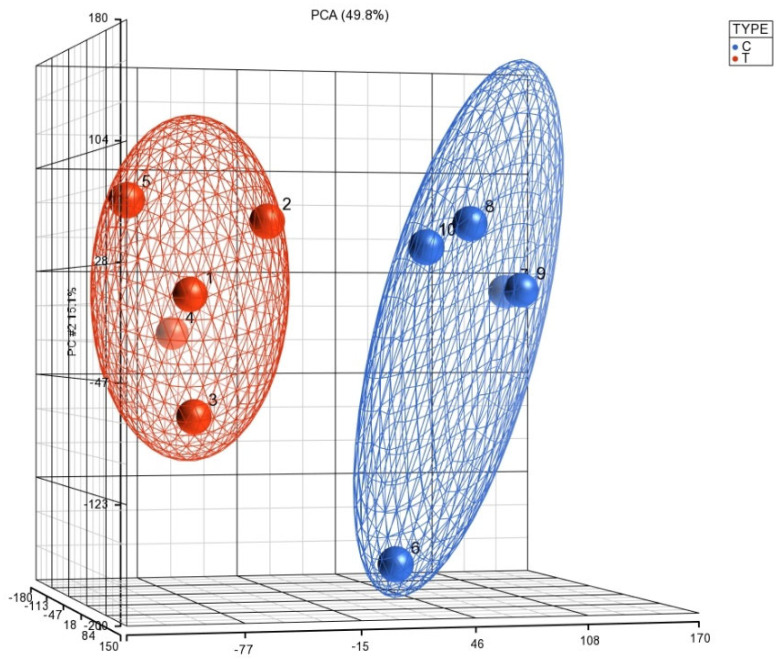
The PCA plot illustrates the distribution of miRNA expression patterns in lung biopsies from COVID-19 positive (T) and negative (C) cases, obtained from postmortem formalin-fixed paraffin-embedded samples using the Affymetrix array system. The *x*-axis represents the expression levels of genes, with negative values indicating downregulation and positive values indicating upregulation. The *y*-axis and *z*-axis denote the percentage of variance explained by the principal components, with PCA 15.1% and PCA 49.8%, respectively. This visualization enables the exploration of overall miRNA expression trends and clustering patterns between COVID-19 positive and negative lung biopsies.

This was supported by Principal Component Analysis (PCA) K-means clustering, which demonstrated the distinct clustering of data between the 5 COVID-19 (+) and 5 COVID-19 (−) biopsy specimens (Figure 2). The 10 most downregulated miRNAs were hsa-miR-30c-5p, hsa-miR-150-5p, hsa-miR-342-3p, hsa-miR-30b-5p, hsa-miR-30a-3p, hsa-miR-99a-5p, hsa-miR-15b-5p, hsa-miR-145-5p, hsa-miR-361-5p, and hsa-miR-125b-5p. The miRNA with the highest differential expression was hsa-miR-30c-5p, with a −5.75965 fold-change in COVID-19 (+) samples. The 10 most upregulated miRNAs were hsa-miR-5703, hsa-miR-5739, hsa-miR-4788, hsa-miR-6086, hsa-miR-4689, hsa-miR-6870-5p, hsa-miR-5195-3p, hsa-miR-6893-5p, hsa-miR-4442, and hsa-miR-642b-3p. The miRNA with the highest differential expression was hsa-miR-642b-3p, with a 5.1684-fold change in COVID-19 (+) samples.

### 3.3. Perturbed mRNA Expression Profiles in COVID-19 Lung Biopsies

**Figure 3:**
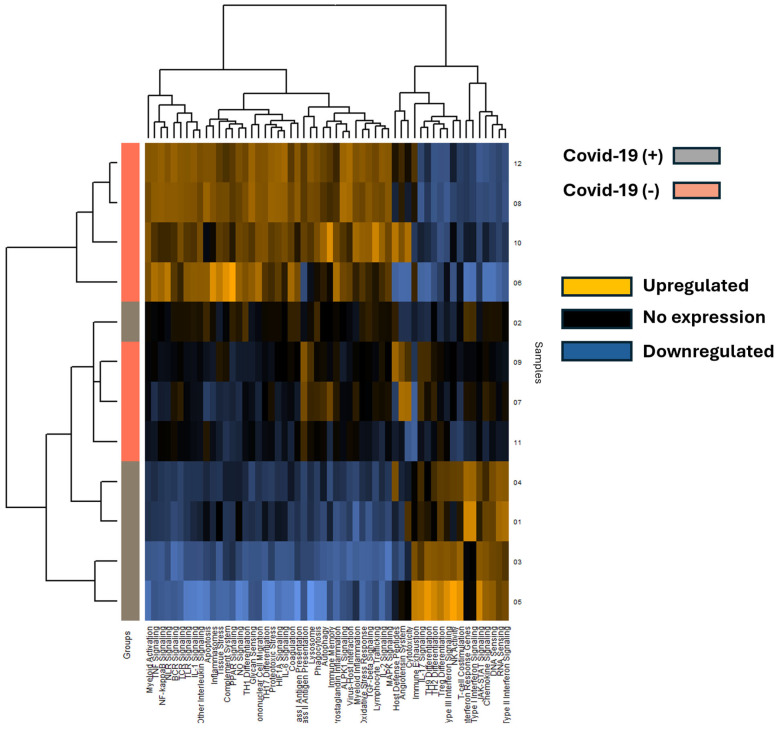
The heatmap displays mRNA pathway scores derived from Nanostring Advanced Analysis, with COVID-19 positive (+) specimens represented by grey rows and COVID-19 negative (−) specimens by salmon-colored rows. Each column represents a differentially expressed mRNA, and patient sample numbers are indicated as rows. Notably, COVID-19 positive sample 2 appears unclustered with other COVID-19 positive samples. Expression levels are color-coded: blue indicates lower expression, black indicates no expression, and gold indicates higher expression.

A total of 653 mRNA transcripts were found to be differentially expressed between the COVID-19 (+) and COVID-19 (−) samples. Interestingly, a greater number of transcripts were downregulated (472) than upregulated (170) in the COVID-19 (+) specimens. The mRNA heatmap of expression was generated using Nanostring Advanced Analysis software, which revealed distinct clustering between COVID-19 (+) and COVID-19 (−) patients (Figure 3). Notably, COVID-19 (+) patient 2 clustered with the COVID-19 (−) specimens, while the added COVID-19 (−) patients 11 and 12 clustered closest and furthest away from the COVID-19 (+) group, respectively. These findings suggest that COVID-19 infection may have a greater impact on downregulating mRNA transcripts in lung tissue. The mRNA transcripts that showed a greater than 2.0 log2-fold change in expression are listed in Table 2, and the top 40 differentially expressed transcripts (both up and down) were further analyzed by miRNA target analysis. 

### 3.4. Differential Gene Expression and Pathway Enrichment of Host Cellular Responses to COVID-19 Infection in Lung

**Figure 4:**
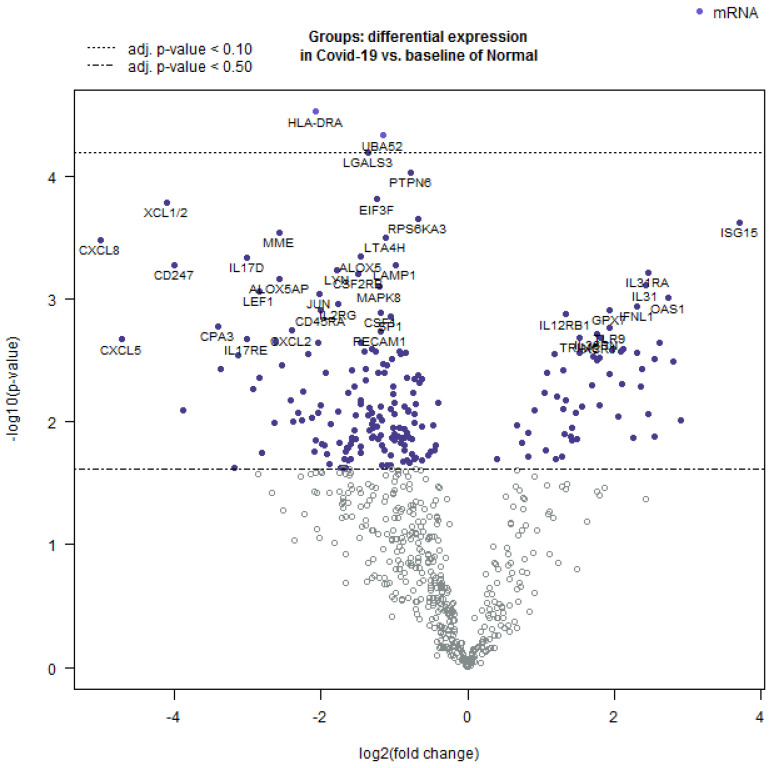
Volcano plot showing the comparison of differentially expressed mRNAs in COVID-19 as compared to normal lung tissue. The log 10-fold change was observed in the variables and the statistical significance was set at *p* < 0.05. The values that are farthest from the center were considered as most significant. The white dots represent markers that are below the threshold and close to 0 and thus represent non-significant fold change.

Further, the Nanostring Advanced Analysis software package was employed to create a volcano plot of differentially expressed transcripts (Figure 4). The highest positive log2-fold differentially expressed mRNA was observed for genes involved in innate immunity, while those with a negative log2-fold differential expression were important for the inflammatory response. 

An undirected and directed global significance scores table was generated using the same package, and three pathways were positively directed in COVID-19 (+) compared to baseline COVID-19 (−), including type III interferon signaling, interferon response genes, and type I interferon signaling (Table 3). Conversely, tissue stress, ALPK1 signaling, and leukotriene and prostaglandin inflammation were negatively directed in COVID-19 (+). 

**Figure 5:**
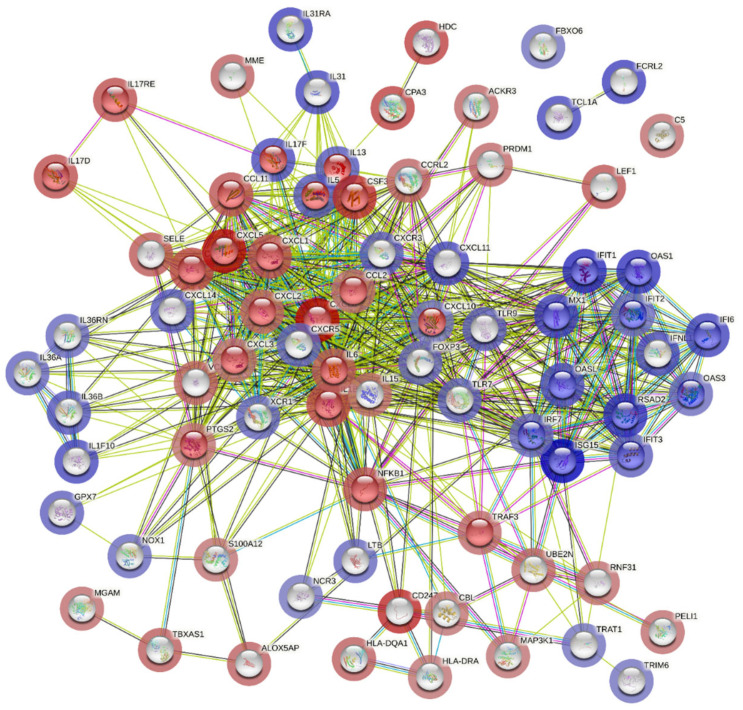
Potential interactions among genes involved in the type 1 interferon signaling pathway using STRING (Search Tool for the Retrieval of Interacting Genes/Proteins). The colored edges reflect different types of protein-protein interactions: red edges indicate interactions curated from known databases, green edges depict interactions predicted through gene fusions, and blue edges represent gene co-occurrence. The proteins jointly contribute to a shared function and do not necessarily physically bind to each other. The nodes are color-coded to represent query proteins and their interaction shells: dark red for the first shell of interaction and light red for the second shell. Similarly, nodes are colored dark and light blue to denote the interaction shells.

Additionally, cluster analysis for pathway enrichment was conducted using the STRING online tool, which revealed that genes involved in the type 1 interferon signaling pathway were highly differentially expressed and associated with upregulated genes in the COVID-19 (+) lung biopsy specimen (Figure 5). 

On the other hand, genes involved in the interleukin-17 signaling pathway correlated with downregulated genes, which corroborated the global significance scores data (Figure 5). These findings provide a molecular signature of the host cellular responses to acute severe COVID-19 infection in lung tissues.

## 4. Discussion

The SARS-CoV-2, a novel coronavirus, is widely recognized to have originated from zoonotic coronaviruses, causing severe pneumonia and lung failure, posing a threat to global public health [17]. Diagnostic kits to test for COVID-19 are currently available, and several antiviral agents, including favipiravir, remdesivir, lopinavir, and ritonavir, have exhibited clinical efficacy against SARS-CoV-2 [18]. Despite the significant progress in developing vaccines and drugs against COVID-19, the mechanisms underlying the pathogenesis of SARS-CoV-2-associated lung disorders in humans remain incompletely understood. Here, we have conducted one of the initial investigations measuring combined miRNA and mRNA expression levels in COVID-19 afflicted lung biopsy tissue. Our study utilizes locally and tissue-specific expressed gene signatures to elucidate the underlying regulatory pathways and networks underlying severe COVID-19 infection and its fatal progression. 

A significant differential expression of microRNAs and mRNAs between COVID-19-infected and non-infected lung tissues was observed in this study. Specifically, we have identified 37 microRNAs that were downregulated and 77 microRNAs that were upregulated in COVID-19-infected tissues. In addition, we found 472 mRNAs that were downregulated and 170 mRNAs that were upregulated in COVID-19 (+) biopsy specimens compared to COVID-19 (−) lung biopsy specimens. Upon further analysis of the differentially expressed mRNA, we observed that the highest and lowest differentially regulated genes were predominantly involved in immune regulation, supporting the notion that immune dysregulation is a crucial factor in the progression of severe COVID-19 infection and death. Of particular interest were two genes, CXCL5 and CXCL8, which play a significant role in neutrophil recruitment and accumulation [19] and have been shown to be dysregulated in the cytokine storm associated with COVID-19 infection [20]. Our dataset demonstrated downregulation of both CXCL5 and CXCL8 in COVID-19 (+) lung biopsy specimens, with these genes having the most negative Log2 fold change difference compared to COVID-19 (−) lung tissues. We also identified miR-23a and miR-23b as significantly downregulated in our samples, which are predicted to target CXCL5, further supporting the role of immune dysregulation in COVID-19 infection. Additionally, we found other mediators of neutrophil recruitment, such as CXCL1 [21], as well as NFKB1 [22], which is important for innate immune response and inflammation activation, to be downregulated in COVID-19 (+) tissues. We also observed patterns of chronic inflammation downregulation in COVID-19 (+) tissues, specifically the downregulation of IL-6, a key gene in promoting chronic inflammation by recruiting monocytic lineages to replace first responder polymorphonuclear neutrophils [23]. Because we used probe alignment systems, COVID-19 viral RNA was not detected and did not influence this analysis.

The innate immune system plays a crucial role in detecting and responding to infections. Dendritic cells, macrophages, and natural killer cells express toll-like receptors (TLRs) that recognize pathogen-associated molecular patterns (PAMPs) and damage-associated molecular patterns (DAMPs) [24]. Activation of TLR pathways leads to the production of pro-inflammatory cytokines, such as interleukin-1 (IL-1), IL-6, tumor necrosis factor-α, and type 1 interferon (IFN-1) [25]. In COVID-19, TLRs, especially TLR7 and TLR8, which bind to single-stranded RNA, are enriched in lung tissues and play critical roles in different stages of the immune response [26]. The TLR pathways promote IFN-1 signaling, which induces the production of pro-inflammatory cytokines and activates the ISG15-mediated antiviral innate response [27]. In COVID-19, the lung tissue response to the viral invasion becomes evident when evaluating the ISG15 pathway. In this study, the highest differentially expressed genes in COVID-19 (+) tissue, including ISG15 itself, IFIT1, RSAD2, OAS1, MX1, OASL, and IFIT3, are all important for the ISG15 pathway’s progression and are part of the lung tissue’s antiviral response. While the ISG15 pathway’s role in antiviral response is established, it is emerging as a key facilitator of local inflammation and poor outcomes in COVID-19 [27,28]. 

Several miRNAs, known to repress and fine-tune this pathway, were identified as downregulated in the miRNA list. For example, hsa-miR-146a-5p exhibited a greater than −3 fold-change in COVID-19 (+) tissue. Predicted miRNA targets, including IFIT1, IFI6, IFIT3, OASL, and OAS3, were all upregulated in the mRNA list and important for the ISG15 pathway’s progression. MiR-146a deficiency is associated with excessive inflammation, as MiR-146a leads to activation of the host antiviral response, and downregulation in chronic inflammatory states such as obesity, diabetes, and hypertension may lead to increased COVID-19 infection severity [29]. Further investigation of miRNAs such as miR-146a and the ISG15 pathway’s modulation is warranted to determine their regulatory role in the antiviral response.

Upon analyzing the datasets, a prominent theme that emerges is the nature of the response elicited. The functional enrichment analysis of STRING revealed that a considerable number of actors involved in pro-inflammatory Th17 response exhibited downregulation, whereas those associated with the T-helper cell subset Th1 response demonstrated upregulation in COVID-19 (+) lung tissue. Although this trend aligns with the current understanding of immunology, several studies conducted on peripheral serum indicate that Th17 cytokines are generally upregulated in systemic COVID-19 infection [30,31,32]. The discrepancy between serum studies and the present data derived from lung biopsy specimens highlights the significance of tissue-level analysis. A closer inspection of the mRNA list unveils another interesting pattern—Th2 supporting cytokines such as IL5 and IL13 are among the most positively upregulated mRNAs in COVID-19 (+) samples. Th2 cell-mediated immune functions are generally classified into two roles: innate response to large extracellular pathogens like helminths and promotion of allergic and atopic immunology [33]. Furthermore, the 11th and 9th most differentially expressed transcripts in COVID-19 (+) biopsy belonged to IL31 and its receptor IL31RA, respectively. IL31 is a potent regulator of atopic response and downstream too, as well as possibly promoting Th2 differentiation. Due to its role in allergic inflammation, IL31 has earned the moniker the “itchy” cytokine and has been linked to inflammatory bowel disease, airway hypersensitivity in asthma, dermatitis, and pulmonary fibrosis [34,35,36]. 

Based on current evidence, there is a strong likelihood that the T-helper cell-mediated immune responses observed in the lung and serum during severe COVID-19 infection may differ significantly. If Th2-mediated allergic reactions are indeed present, this could lead to significant implications for the prevention and treatment of severe COVID-19. To gain deeper insight into this phenomenon, miRNA datasets should be examined. Specifically, the almost 3-fold downregulation of hsa-let-7g-5p, which is predicted to target IL13, is noteworthy. The Let-7g family is known to modulate inflammation and has been shown to directly reduce allergic airway inflammation by binding IL13. Recent studies have hypothesized that IL13 may be a driver of COVID-19 severity [37]. Other miRNAs and mRNA targets, such as downregulated hsa-miR-125b-5p and upregulated IL31, as well as downregulated hsa-miR-145-5p and upregulated IL31a, are also present. It is plausible that the development of diffuse alveolar damage and acute respiratory distress syndrome during severe COVID-19 infection could be partially allergy-mediated through dysregulation, which necessitates further research.

Taken together, our findings highlight the crucial role of immune dysregulation in the pathogenesis of severe COVID-19 infection in lungs and provide valuable insights into potential therapeutic targets for mitigating the deleterious effects of COVID-19.

## 5. Limitations

### 5.1. Limited Information on Lung Sample Location 

The present study lacks data regarding the specific location within the lung from which the samples were obtained. While acknowledging the importance of such information, particularly in the context of SARS-CoV-2 infection dynamics, this study was constrained by the available resources. Nevertheless, future investigations could benefit from incorporating this detail to enhance the understanding of viral tropism and host response.

### 5.2. Low Sample Size 

Another constraint of the present study is the relatively small sample size. Increasing the sample size could enhance the statistical power of the analyses and provide more robust conclusions. Efforts to expand sample collection should be pursued in future research endeavors to further validate our findings.

### 5.3. Selection of Control Group 

The choice of control group, comprising individuals with various non-COVID-19-related conditions like atherosclerosis and diabetes could also influence the immune signaling and could affect the results. While this selection allowed for comparisons across different inflammatory contexts, it may introduce confounding factors, particularly considering the dysregulation of pro-inflammatory signals and immune-related proteins in affected tissues. 

## 6. Conclusions

Immune dysregulation emerges as a crucial factor in the progression of severe COVID-19 infection and death, with genes involved in immune regulation showing the highest differential regulation. This study sheds light on the underlying mechanisms of severe COVID-19 infection and its fatal progression by analyzing locally and tissue-specific expressed gene signatures. The findings reveal significant differential expression of microRNAs and mRNAs in COVID-19-infected lung tissues compared to non-infected tissues. The study also identifies downregulation of key genes involved in neutrophil recruitment and accumulation, as well as chronic inflammation, suggesting their role in COVID-19 infection. The innate immune response, toll-like receptor pathways, and the ISG15 pathway are implicated in the lung tissue’s antiviral response to COVID-19. Dysregulated miRNAs in the ISG15 pathway and Th2-mediated immune responses may have implications for the severity of COVID-19 infection and the development of allergic reactions. Further research is needed to explore the regulatory roles of miRNAs and the modulation of immune responses in COVID-19.

## Figures and Tables

**Table 1:** Demograhic details of the COVID-19 positive and negative samples.

No.	Disease Status	Date of Admission	Date of Death	Gender	AGE	Cause of Death
1	Covid + ve	9/8/2020	9/18/2020	MALE	45	COVID-19 Pulmonary injury
2	Covid + ve	11/22/2020	11/24/2020	MALE	65	COVID-19 Pneumonia
3	Covid + ve	9/20/2020	10/4/2020	MALE	51	COVID-19 Pneumonia
4	Covid + ve	7/23/2020	7/25/2020	FEMALE	40	COVID-19 Pneumonia
5	Covid + ve	7/12/2020	8/10/2020	MALE	80	COVID-19 Pneumonia
6	Covid − ve	8/17/2020	8/24/2020	MALE	90	Atherosclerotic cardiovascular disease
7	Covid − ve	2/14/2021	2/15/2021	MALE	52	Cerebrovascular accident secondary to hypertensive cardiovascular disease
8	Covid − ve	2/15/2021	2/24/2021	FEMALE	72	Atherosclerotic cardiovascular disease with diabetes mellitus and end-stage renal disease
9	Covid − ve	3/25/2021	3/25/2021	FEMALE	100	Hypertensive cardiovascular disease
10	Covid − ve	2/15/2021	2/24/2021	FEMALE	72	Atherosclerotic cardiovascular disease with diabetes mellitus and end-stage renal disease
11	Covid − ve	4/13/2021	4/13/2021	FEMALE	54	Cardiac arrhythmia due to cardiomegaly due to hypertension. Diabetes mellitus as a cofactor
12	Covid − ve	9/13/2020	9/15/2020	MALE	33	Midbrain hemorrhage due to hypertensive and atherosclerotic cardiovascular disease

**Table 2:** List of differentially expressed mRNA with log2 fold change, standard error, confidence, and significant limits along with the associated gene sets.

mRNAs Involved	Log2 Fold Change	Std Error (log2)	Lower Confidence Limit (log2)	Upper Confidence Limit (log2)	*p*-Value	Gene Sets
CXCL8-mRNA	−5.01	0.94	−6.85	−3.17	0.000331	Chemokine signaling, myeloid activation, NF-kappaB signaling, NLR signaling, RNA sensing, tissue stress, TLR signaling
CXCL5-mRNA	−4.72	1.15	−6.97	−2.47	0.00213	Chemokine signaling, TNF signaling
XCL1/2-mRNA	−4.1	0.702	−5.48	−2.73	0.000163	Chemokine signaling, mononuclear cell migration
CD247-mRNA	−4	0.798	−5.56	−2.44	0.000528	NK activity, TCR signaling
CSF3-mRNA	−3.87	1.17	−6.17	−1.57	0.008	JAK-STAT signaling, other interleukin signaling
CPA3-mRNA	−3.4	0.799	−4.97	−1.84	0.00167	Angiotensin system
HDC-mRNA	−3.37	0.898	−5.13	−1.61	0.00374	Myeloid inflammation
CCL20-mRNA	−3.18	1.19	−5.52	−0.84	0.0239	Chemokine signaling, mononuclear cell migration, TNF signaling
IL1B-mRNA	−3.12	0.797	−4.69	−1.56	0.00287	DNA sensing, glycan sensing, IL-1 signaling, inflammasomes, NF-kappaB signaling, NLR signaling, Th17 differentiation, TNF signaling
IL17RE-mRNA	−3.01	0.733	−4.44	−1.57	0.00214	IL-17 signaling
IL17D-mRNA	−3	0.588	−4.15	−1.85	0.000458	JAK-STAT signaling
CXCL1-mRNA	−2.92	0.828	−4.54	−1.3	0.00545	Chemokine signaling, myeloid activation, NF-kappaB signaling, NLR signaling, TNF signaling
CCL11-mRNA	−2.85	1.1	−5	−0.69	0.0269	Chemokine signaling, mononuclear cell migration
LEF1-mRNA	−2.84	0.607	−4.03	−1.65	0.000868	TCR signaling
NFKB1-mRNA	−2.83	0.772	−4.34	−1.32	0.00433	BCR signaling, DNA sensing, Glycan sensing, Inflammasomes, myeloid activation, NF-kappaB signaling, NLR signaling, RNA sensing, TCR signaling, Th1 differentiation, TLR signaling, TNF signaling, Type I interferon signaling
SELE-mRNA	−2.8	0.99	−4.74	−0.86	0.018	Lymphocyte trafficking, TNF signaling
TBXAS1-mRNA	−2.66	1.11	−4.85	−0.48	0.0379	Coagulation
IL6-mRNA	−2.64	0.835	−4.28	−1	0.0101	DNA sensing, HIF1A signaling, IL-6 signaling, JAK-STAT signaling, mononuclear cell migration, myeloid activation, NLR signaling, Th17 differentiation, Th2 differentiation, tissue stress, TNF signaling
HLA-DQA-mRNA	−2.61	0.641	−3.87	−1.36	0.00223	Lymphocyte trafficking, MHC Class II antigen presentation, phagocytosis, T cell costimulation, TCR signaling, type II interferon signaling
ALOX5AP-mRNA	−2.57	0.532	−3.62	−1.53	0.000687	Leukotriene and prostaglandin inflammation, myeloid inflammation
CXCL14-mRNA	2.06	0.639	0.806	3.31	0.00915	Chemokine signaling
IL13-mRNA	2.08	0.525	1.05	3.1	0.0027	IL-1 signaling, JAK-STAT signaling, myeloid activation, myeloid inflammation, Th2 differentiation
IL5-mRNA	2.1	0.585	0.957	3.25	0.00488	IL-2 signaling, JAK-STAT signaling, TCR signaling, Th2 differentiation
IL36B-mRNA	2.12	0.531	1.08	3.16	0.00253	IL-1 signaling
OASL-mRNA	2.25	0.753	0.775	3.73	0.0136	Interferon response genes, type I interferon signaling, type II interferon signaling
IFNL1-mRNA	2.3	0.511	1.3	3.3	0.00115	JAK-STAT signaling, other interleukin signaling, type III interferon signaling
IL17F-mRNA	2.3	0.583	1.16	3.45	0.00273	IL-17 signaling, Th17 differentiation
IL1F10-mRNA	2.36	0.664	1.06	3.66	0.00521	IL-1 signaling
TCL1A-mRNA	2.37	0.63	1.13	3.61	0.00372	
IL31-mRNA	2.42	0.51	1.42	3.42	0.00078	IL-6 signaling
CXCL11-mRNA	2.42	1.04	0.383	4.46	0.0422	Chemokine signaling
IL31RA-mRNA	2.46	0.501	1.48	3.45	0.000608	IL-6 signaling, myeloid activation
IFI6-mRNA	2.46	0.754	0.981	3.94	0.00857	Type I interferon signaling
MX1-mRNA	2.54	0.654	1.25	3.82	0.00307	Interferon response genes, type I interferon signaling
FCRL2-mRNA	2.54	0.847	0.884	4.2	0.0133	
IFNA1/13-mRNA	2.61	0.642	1.35	3.87	0.00228	DNA sensing, JAK-STAT signaling, NLR signaling, RNA sensing, TLR signaling
OAS1-mRNA	2.74	0.597	1.57	3.91	0.000984	Interferon response genes, NLR signaling, type I interferon signaling, type II interferon signaling
RSAD2-mRNA	2.8	0.727	1.37	4.22	0.00323	type I interferon signaling
IFIT1-mRNA	2.91	0.914	1.12	4.7	0.00979	Interferon response genes, type I interferon signaling
ISG15-mRNA	3.7	0.666	2.4	5.01	0.000239	Interferon response genes, RNA sensing, type I interferon signaling

**Table 3:** The Nanostring undirected and directed global significance scores table showcases the global significance scores and directed global significance scores for each sample, as defined in the accompanying heatmaps. The global significance score is derived from the square root of the mean squared t-statistic for the genes within a gene set, utilizing the t-statistics obtained from the linear regression that underlies our differential expression analysis. Similarly, the directed global significance score is calculated from the square root of the mean signed squared t-statistic for the genes within a gene set, utilizing the same t-statistics from the linear regression.

Pathways/Gene Sets Involved	Undirected Groups: Differential Expression in COVID-19 vs. Baseline of Normal	Directed Groups: Differential Expression in COVID-19 vs. Baseline of Normal
Type III Interferon Signaling	2.469	2.081
Interferon Response Genes	2.876	1.654
Type I Interferon Signaling	2.743	0.54
Immune Exhaustion	1.794	−0.737
Treg Differentiation	1.94	−1.234
Th9 Differentiation	1.563	−1.272
MHC Class I Antigen Presentation	1.964	−1.406
IL-6 Signaling	2.866	−1.414
Type II Interferon Signaling	2.735	−1.446
Th1 Differentiation	2.328	−1.498
Host Defense Peptides	1.52	−1.52
DNA Sensing	2.481	−1.523
Other Interleukin Signaling	2.218	−1.539
T-cell Costimulation	2.46	−1.545
Th17 Differentiation	2.619	−1.684
Th2 Differentiation	2.826	−1.693
Oxidative Stress Response	2.372	−1.769
JAK-STAT Signaling	2.938	−1.791
NLR Signaling	2.488	−1.811
NO Signaling	1.908	−1.828
Proteotoxic Stress	2.301	−1.849
IL-1 Signaling	2.758	−1.903
Inflammasomes	2.134	−1.909
NK Activity	2.575	−1.912
Autophagy	2.238	−1.92
Chemokine Signaling	2.651	−1.946
Cytotoxicity	1.983	−1.974
RNA Sensing	2.974	−1.982
HIF1A Signaling	2.208	−1.994
Myeloid Inflammation	2.651	−2.1
Apoptosis	2.144	−2.103
Complement System	2.164	−2.11
Phagocytosis	2.249	−2.164
NF-kappaB Signaling	2.535	−2.169
Lymphocyte Trafficking	2.325	−2.224
IL-17 Signaling	2.841	−2.225
TLR Signaling	2.706	−2.225
IL-2 Signaling	2.679	−2.255
BCR Signaling	2.636	−2.293
Glycan Sensing	2.572	−2.317
Coagulation	2.455	−2.337
Myeloid Activation	2.594	−2.393
Lysosome	2.503	−2.41
MAPK Signaling	2.816	−2.472
Mononuclear Cell Migration	2.613	−2.478
TCR Signaling	2.836	−2.495
TNF Signaling	2.691	−2.623
PPAR Signaling	2.663	−2.663
Angiotensin System	2.721	−2.709
Virus-Host Interaction	2.786	−2.736
TGF-beta Signaling	2.769	−2.768
Immune Memory	3.155	−2.987
MHC Class II Antigen Presentation	3.03	−3.011
Tissue Stress	3.146	−3.146
ALPK1 Signaling	3.419	−3.18
Leukotriene and Prostaglandin Inflammation	3.382	−3.218

## Data Availability

No new datasets were created.

## Supplementary Materials

The following supporting information can be downloaded at: https://www.mdpi.com/article/10.3390/v16060853/s1, Table S1: List of reference genes included in the NanoString’s nCounter^®^ Host Reponse Panel, Table S2: Showing the summary of Pathways, Cell Types, and Functional Themes provided by the NanoString’s nCounter^®^ Host Reponse Panel, Table S3: Showing the differentially expressed miRNAs in COVID-19 (+) and COVID-19 (−) lung biopsy specimen. The *p*-value of 0.05 was taken as statistically significant.
