# Supplementary material for: Gene Regulatory Network Analysis of Post-Mortem Lungs Unveils Novel Insights into COVID-19 Pathogenesis
Source: Viruses. 2024 May 27;16(6):853. doi: 10.3390/v16060853 (PMC11209433; doi:10.3390/v16060853)
Supplement: Supplementary file 1 [file viruses-16-00853-s001.zip › viruses-3017085-supplementary.pdf]

Supplementary Materials

Table S1. List of reference genes included in the NanoString's nCounter® Host Reponse Panel.

S.No.	Genes
1	ABCF1
2	ALAS1
3	GUSB
4	HPRT1
5	MRPS7
6	NMT1
7	NRDE2
8	OAZ1
9	PGK1
10	SDHA
11	STK11IP
12	TBP

Table S2. Showing the summary of Pathways, Cell Types, and Functional Themes provided by the NanoString's nCounter® Host Reponse Panel

Pathway Summary	# Genes
ALPK1 Signaling	7
Angiotensin System	12
Apoptosis	27
Autophagy	15
BCR Signaling	55
Chemokine Signaling	75
Coagulation	33
Complement System	23
Cytotoxicity	11
DNA Sensing	44
Glycan Sensing	38
HIF1A Signaling	21
Host Defense Peptides	15
IL-1 Signaling	45
IL-17 Signaling	20
IL-2 Signaling	29
IL-6 Signaling	16
Immune Exhaustion	17
Immune Memory	9
Inflammasomes	22
Interferon Response Genes	24
JAK-STAT Signaling	71

Leukotriene and Prostaglandin Inflammation	13
Lymphocyte Trafficking	25
Lysosome	27
MAPK Signaling	24
MHC Class I Antigen Presentation	34
MHC Class II Antigen Presentation	17
Mononuclear Cell Migration	54
Myeloid Activation	167
Myeloid Inflammation	27
NF-kappaB Signaling	75
NK Activity	32
NLR Signaling	95
NO Signaling	9
Other Interleukin Signaling	77
Oxidative Stress Response	44
Phagocytosis	30
PPAR Signaling	7
Proteotoxic Stress	28
RNA Sensing	56
T-cell Costimulation	28
TCR Signaling	92
TGF-beta Signaling	28
Th1 Differentiation	19
Th17 Differentiation	36
Th2 Differentiation	18
Th9 Differentiation	10
Tissue Stress	7
TLR Signaling	76
TNF Signaling	72
Treg Differentiation	14
Type I Interferon Signaling	68
Type II Interferon Signaling	41
Type III Interferon Signaling	10
Virus-Host Interaction	14
Cell Type Summary	# Genes
B-cells	9
CD45	1
CD8 T cells	2
Cytotoxic cells	10
DC	3
Exhausted CD8	4
Macrophages	4
Mast cells	4
Neutrophils	7
NK CD56dim cells	3

NK cells	2
T-cells	6
Th1 cells	1
Treg	1
Functional Theme Summary	# Genes
Adaptive Immune Response	483
Homeostasis	282
Host Susceptibility	26
Innate Immune Cell Activation	567
Interferon Response	288

Table S3. Showing the differentially expressed miRNAs in COVID-19 (+) and COVID-19 (-) lung biopsy specimen. The p-value of 0.05 was taken as statistically significant.

**Probe set ID**	**Species Scientific Name**	**Transcript ID(Array Design)**	**p-value(T vs. C)**	**Fold-Change(T vs. C)**	**Fold-Change(T vs. C) (Description)**
MIMAT0000244_st	Homo sapiens	hsa-miR-30c-5p	0.00779607	-5.75965	T down vs C
MIMAT0000451_st	Homo sapiens	hsa-miR-150-5p	0.0051347	-5.09846	T down vs C
MIMAT0000753_st	Homo sapiens	hsa-miR-342-3p	0.000820238	-4.89208	T down vs C
MIMAT0000420_st	Homo sapiens	hsa-miR-30b-5p	0.00889431	-4.75996	T down vs C
MIMAT0000088_st	Homo sapiens	hsa-miR-30a-3p	0.00128903	-4.56757	T down vs C
MIMAT0000097_st	Homo sapiens	hsa-miR-99a-5p	0.00333297	-4.49274	T down vs C
MIMAT0000417_st	Homo sapiens	hsa-miR-15b-5p	0.0227925	-4.36901	T down vs C
MIMAT0000437_st	Homo sapiens	hsa-miR-145-5p	0.0159752	-3.98813	T down vs C
MIMAT0000703_st	Homo sapiens	hsa-miR-361-5p	0.00164426	-3.97822	T down vs C
MIMAT0000423_st	Homo sapiens	hsa-miR-125b-5p	0.00129893	-3.96122	T down vs C
MIMAT0000086_st	Homo sapiens	hsa-miR-29a-3p	0.00096328	-3.8483	T down vs C
MIMAT0000419_st	Homo sapiens	hsa-miR-27b-3p	0.0125919	-3.81779	T down vs C

U34_st	Homo sapiens	U34	0.0122249	-3.60867	T down vs C
MIMAT0000232_st	Homo sapiens	hsa-miR-199a-3p	0.0342405	-3.44361	T down vs C
MIMAT0004563_st	Homo sapiens	hsa-miR-199b-3p	0.0342405	-3.44361	T down vs C
MIMAT0000418_st	Homo sapiens	hsa-miR-23b-3p	0.0213144	-3.41425	T down vs C
MIMAT0001340_st	Homo sapiens	hsa-miR-423-3p	0.0123837	-3.37476	T down vs C
U29_st	Homo sapiens	U29	0.0120609	-3.34893	T down vs C
MIMAT0000084_st	Homo sapiens	hsa-miR-27a-3p	0.0254802	-3.32941	T down vs C
MIMAT0000098_st	Homo sapiens	hsa-miR-100-5p	0.0129771	-3.24286	T down vs C
ACA44_s_st	Homo sapiens	ACA44	0.00910841	-3.23556	T down vs C
ENSG00000252840_s_st	Homo sapiens	ENSG00000252840	0.00910841	-3.23556	T down vs C
MIMAT0000081_st	Homo sapiens	hsa-miR-25-3p	0.0114454	-3.19382	T down vs C
MIMAT0003322_st	Homo sapiens	hsa-miR-652-3p	0.00854433	-3.17227	T down vs C
MIMAT0002809_st	Homo sapiens	hsa-miR-146b-5p	0.0123015	-3.0781	T down vs C
MIMAT0000449_st	Homo sapiens	hsa-miR-146a-5p	0.0123919	-3.05051	T down vs C
MIMAT0000278_st	Homo sapiens	hsa-miR-221-3p	0.0183222	-2.97784	T down vs C
MIMAT0000728_st	Homo sapiens	hsa-miR-375	0.00545275	-2.96197	T down vs C
MIMAT0003393_st	Homo sapiens	hsa-miR-425-5p	0.004824	-2.92163	T down vs C
MIMAT0000440_st	Homo sapiens	hsa-miR-191-5p	0.00841381	-2.90219	T down vs C
MIMAT0000414_st	Homo sapiens	hsa-let-7g-5p	0.0104919	-2.90068	T down vs C
MIMAT0000078_st	Homo sapiens	hsa-miR-23a-3p	0.0150191	-2.89633	T down vs C
MIMAT0000689_st	Homo sapiens	hsa-miR-99b-5p	0.02095	-2.83642	T down vs C
MIMAT0009447_st	Homo sapiens	hsa-miR-1972	0.00155372	-2.81157	T down vs C
MIMAT0010214_st	Homo sapiens	hsa-miR-151b	0.00571411	-2.76076	T down vs C
MIMAT0022272_st	Homo sapiens	hsa-miR-664b-3p	0.0192134	-2.62046	T down vs C
MIMAT0000280_st	Homo sapiens	hsa-miR-223-3p	0.00784884	-2.57209	T down vs C
MIMAT0027514_st	Homo sapiens	hsa-miR-6807-5p	3.89E-05	2.53442	T up vs C

MIMAT0027450_st	Homo sapiens	hsa-miR-6775-5p	0.00906079	2.55976	T up vs C
MIMAT0019928_st	Homo sapiens	hsa-miR-4773	0.0016936	2.56721	T up vs C
MIMAT0019978_st	Homo sapiens	hsa-miR-4800-5p	0.00107145	2.5728	T up vs C
MIMAT0016889_st	Homo sapiens	hsa-miR-4327	0.00726464	2.57621	T up vs C
MIMAT0015087_st	Homo sapiens	hsa-miR-514b-5p	0.00478895	2.57954	T up vs C
MI0020366_s_st	Homo sapiens	hsa-mir-6089-1	0.000530889	2.58178	T up vs C
MI0023563_s_st	Homo sapiens	hsa-mir-6089-2	0.000530889	2.58178	T up vs C
MIMAT0028119_st	Homo sapiens	hsa-miR-7111-5p	0.00147168	2.58242	T up vs C
MIMAT0023710_st	Homo sapiens	hsa-miR-6085	0.0175388	2.58591	T up vs C
MIMAT0022693_st	Homo sapiens	hsa-miR-204-3p	0.000756128	2.58824	T up vs C
MIMAT0030414_st	Homo sapiens	hsa-miR-4433b-3p	0.000451336	2.61494	T up vs C
MIMAT0027387_st	Homo sapiens	hsa-miR-6743-5p	0.00602158	2.61563	T up vs C
MIMAT0026486_st	Homo sapiens	hsa-miR-328-5p	0.00607654	2.62207	T up vs C
MIMAT0016901_st	Homo sapiens	hsa-miR-4271	0.0211995	2.62737	T up vs C
MIMAT0031012_st	Homo sapiens	hsa-miR-8085	0.000628915	2.66353	T up vs C
MIMAT0019043_st	Homo sapiens	hsa-miR-2392	0.00924498	2.66487	T up vs C
MIMAT0024599_st	Homo sapiens	hsa-miR-6126	9.08E-05	2.678	T up vs C
MIMAT0018068_st	Homo sapiens	hsa-miR-3648	0.026009	2.67961	T up vs C
MIMAT0027620_st	Homo sapiens	hsa-miR-6769b-5p	0.0107804	2.68295	T up vs C
MIMAT0027412_st	Homo sapiens	hsa-miR-6756-5p	0.00993804	2.68708	T up vs C
MIMAT0027660_st	Homo sapiens	hsa-miR-6880-5p	0.00651452	2.69802	T up vs C
MIMAT0027398_st	Homo sapiens	hsa-miR-6749-5p	0.00169593	2.70426	T up vs C
MIMAT0022691_st	Homo sapiens	hsa-miR-197-5p	0.00696774	2.70983	T up vs C
MIMAT0016907_st	Homo sapiens	hsa-miR-4281	0.00499818	2.72882	T up vs C
MIMAT0025476_st	Homo sapiens	hsa-miR-6510-5p	9.82E-06	2.76967	T up vs C
MIMAT0024782_st	Homo sapiens	hsa-miR-6165	0.00734075	2.78008	T up vs C

MIMAT0027558_st	Homo sapiens	hsa-miR-6829-5p	0.00273428	2.78419	T up vs C
MIMAT0027436_st	Homo sapiens	hsa-miR-6768-5p	0.0155299	2.7914	T up vs C
MIMAT0019743_st	Homo sapiens	hsa-miR-4667-5p	0.000886903	2.80467	T up vs C
MIMAT0021128_st	Homo sapiens	hsa-miR-5196-5p	0.00302993	2.82337	T up vs C
MIMAT0027658_st	Homo sapiens	hsa-miR-6879-5p	0.0155111	2.84337	T up vs C
MIMAT0030987_st	Homo sapiens	hsa-miR-8060	0.000708309	2.86911	T up vs C
MIMAT0019073_st	Homo sapiens	hsa-miR-4534	0.0162475	2.88306	T up vs C
MIMAT0027524_st	Homo sapiens	hsa-miR-6812-5p	0.0188905	2.89736	T up vs C
MIMAT0027404_st	Homo sapiens	hsa-miR-6752-5p	0.00435241	2.91286	T up vs C
MI0014182_st	Homo sapiens	hsa-mir-3154	0.000184849	2.9214	T up vs C
MIMAT0015005_st	Homo sapiens	hsa-miR-3137	3.64E-05	2.92944	T up vs C
MIMAT0027516_st	Homo sapiens	hsa-miR-6808-5p	0.00572864	2.93884	T up vs C
MIMAT0024597_st	Homo sapiens	hsa-miR-6124	0.00866458	2.93965	T up vs C
MIMAT0016878_st	Homo sapiens	hsa-miR-4257	0.000213241	2.95819	T up vs C
MIMAT0015038_st	Homo sapiens	hsa-miR-3164	0.000659677	2.96629	T up vs C
MIMAT0028117_st	Homo sapiens	hsa-miR-7110-5p	0.00110187	2.96952	T up vs C
MIMAT0018981_st	Homo sapiens	hsa-miR-4459	0.0152655	3.03585	T up vs C
MIMAT0027377_st	Homo sapiens	hsa-miR-6738-5p	0.00224091	3.05261	T up vs C
MIMAT0027494_st	Homo sapiens	hsa-miR-6797-5p	0.00661162	3.05312	T up vs C
MIMAT0018104_st	Homo sapiens	hsa-miR-3679-5p	0.0016725	3.05968	T up vs C
MIMAT0015052_st	Homo sapiens	hsa-miR-3175	0.00494965	3.06188	T up vs C
MIMAT0027674_st	Homo sapiens	hsa-miR-6887-5p	0.00248618	3.08499	T up vs C
MIMAT0023701_st	Homo sapiens	hsa-miR-6076	0.00144915	3.08579	T up vs C
MIMAT0024610_st	Homo sapiens	hsa-miR-6127	0.00350322	3.12797	T up vs C
MIMAT0027426_st	Homo sapiens	hsa-miR-6763-5p	0.00336943	3.14849	T up vs C
MIMAT0027646_st	Homo sapiens	hsa-miR-6873-5p	0.000469586	3.16764	T up vs C

MIMAT0018987_st	Homo sapiens	hsa-miR-4463	0.00274623	3.17578	T up vs C
MIMAT0019035_st	Homo sapiens	hsa-miR-4499	0.000653507	3.18257	T up vs C
MIMAT0027496_st	Homo sapiens	hsa-miR-6798-5p	0.00310264	3.23372	T up vs C
MIMAT0018943_st	Homo sapiens	hsa-miR-4428	0.00712091	3.24741	T up vs C
MIMAT0016900_st	Homo sapiens	hsa-miR-4270	0.00563253	3.2502	T up vs C
MIMAT0027682_st	Homo sapiens	hsa-miR-6891-5p	0.000504234	3.47554	T up vs C
MIMAT0015028_st	Homo sapiens	hsa-miR-3154	0.00290703	3.47681	T up vs C
MIMAT0027464_st	Homo sapiens	hsa-miR-6782-5p	0.00303651	3.49903	T up vs C
MIMAT0028211_st	Homo sapiens	hsa-miR-7150	0.0029549	3.55933	T up vs C
MIMAT0005929_st	Homo sapiens	hsa-miR-1275	0.00349958	3.57815	T up vs C
MIMAT0027572_st	Homo sapiens	hsa-miR-6780b-5p	2.79E-05	3.61738	T up vs C
MIMAT0027504_st	Homo sapiens	hsa-miR-6802-5p	0.0207247	3.6366	T up vs C
MIMAT0018961_st	Homo sapiens	hsa-miR-4443	0.00691102	3.64809	T up vs C
MIMAT0027654_st	Homo sapiens	hsa-miR-6877-5p	0.000797402	3.70387	T up vs C
MIMAT0022496_st	Homo sapiens	hsa-miR-5703	0.00118643	3.70807	T up vs C
MIMAT0023116_st	Homo sapiens	hsa-miR-5739	3.39E-05	3.72099	T up vs C
MIMAT0019958_st	Homo sapiens	hsa-miR-4788	0.00559632	3.78632	T up vs C
MIMAT0023711_st	Homo sapiens	hsa-miR-6086	1.37E-05	3.85342	T up vs C
MIMAT0019778_st	Homo sapiens	hsa-miR-4689	0.00156871	3.86747	T up vs C
MIMAT0027640_st	Homo sapiens	hsa-miR-6870-5p	0.000632641	3.95504	T up vs C
MIMAT0021127_st	Homo sapiens	hsa-miR-5195-3p	0.000346523	4.31313	T up vs C
MIMAT0027686_st	Homo sapiens	hsa-miR-6893-5p	0.000434091	4.45657	T up vs C
MIMAT0018960_st	Homo sapiens	hsa-miR-4442	0.00512066	4.96925	T up vs C
MIMAT0018444_st	Homo sapiens	hsa-miR-642b-3p	0.000287101	5.1684	T up vs C
